# Crystal structure of luliconazole

**DOI:** 10.1107/S2056989024011812

**Published:** 2025-01-01

**Authors:** Anna Ben, Lilianna Chęcińska

**Affiliations:** a University of Lodz Doctoral School of Exact and Natural Sciences Narutowicza 68 90-136 Łódź Poland; b University of Lodz Faculty of Chemistry Pomorska 163/165 90-236 Łódź Poland; Katholieke Universiteit Leuven, Belgium

**Keywords:** luliconazole, crystal structure, Hirshfeld surface analysis

## Abstract

The crystal structure of luliconazole {LCZ; C_14_H_9_Cl_2_N_3_S_2_; systematic name: (*E*)-[(4*R*)-4-(2,4-di­chloro­phen­yl)-1,3-di­thio­lan-2-yl­idene](1*H*-imidazol-1-yl)aceto­nitrile} is reported. In the mol­ecule of the title compound, the di­thiol­ane ring adopts an envelope conformation, while the di­chloro­phenyl ring exhibits disorder. In the crystal packing of luliconazole, only two inter­molecular C–H⋯N hydrogen bonds are observed. Hirshfeld surface analysis reveals that the most dominant contacts are H⋯N/N⋯H, H⋯Cl/Cl⋯H, H⋯H and C⋯H/H⋯C.

## Abstract 2

The crystal structure of luliconazole (LCZ) has been determined. The di­thiol­ane ring adopts an envelope conformation. In the crystal, two inter­molecular C—H⋯N hydrogen bonds are observed.

## 1. Chemical context

Luliconazole {LCZ; C_14_H_9_Cl_2_N_3_S_2_; CAS No. 187164-19-8; systematic name: (*E*)-[(4*R*)-4-(2,4-di­chloro­phen­yl)-1,3-di­thio­lan-2-yl­idene](1*H*-imidazol-1-yl)aceto­nitrile} is a prominent anti­fungal agent and belongs to the azole class of drugs, specifically imidazole derivatives. The compound possesses a distinctive chemical structure, enhanced by the incorporation of an imidazole moiety into the ketene di­thio­acetate framework. This structural modification retains the broad anti­fungal spectrum of imidazole drugs, while achieving high potency against filamentous fungi, including dermatophytes (Koga *et al.*, 2012 ▸).
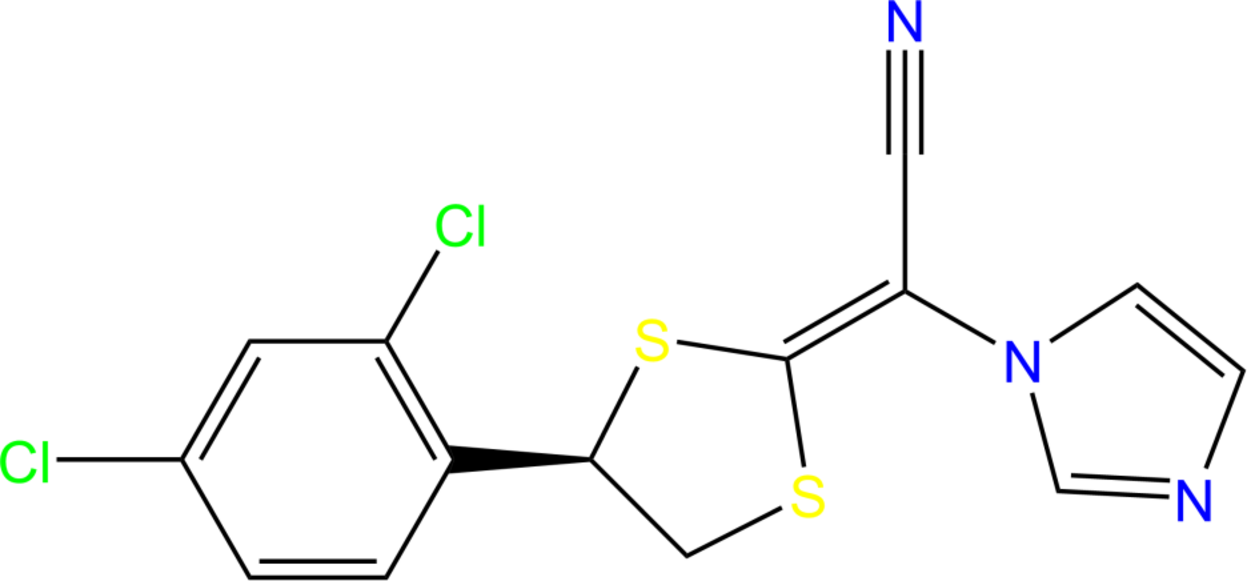


Luliconazole, as the *R*-enanti­omer, exhibits significantly greater anti­fungal activity compared to lanoconazole, which exists as a racemic mixture (Deepshikha & Subhash, 2014 ▸). The key distinction between these two compounds lies in their stereochemistry: lanoconazole is a racemic compound, while luliconazole is the pure *R*-enanti­omer. Inter­estingly, the *S*-enanti­omers of both compounds are inactive as anti­fungal agents, making LCZ inherently more potent (Niwano *et al.*, 1998 ▸).

This article provides a detailed structural analysis of the pure drug luliconazole, which has not previously been reported in the literature.

## 2. Structural commentary

**Figure 1:**
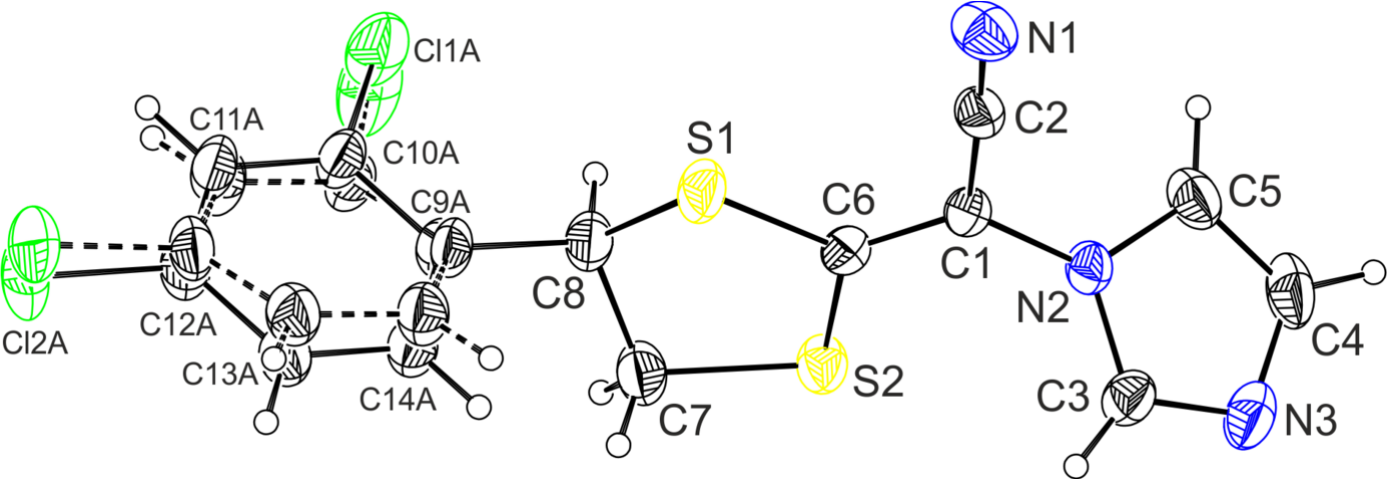
The mol­ecular structure of LCZ with the atom-numbering scheme. The disordered components A and B of the di­chloro­phenyl ring have equal site-occupancies (1/2). Component A is drawn using unbroken lines while component B is drawn using dashed lines without labels. Displacement ellipsoids are drawn at the 30% probability level and H atoms are shown as small spheres of arbitrary radii.

**Figure 2:**
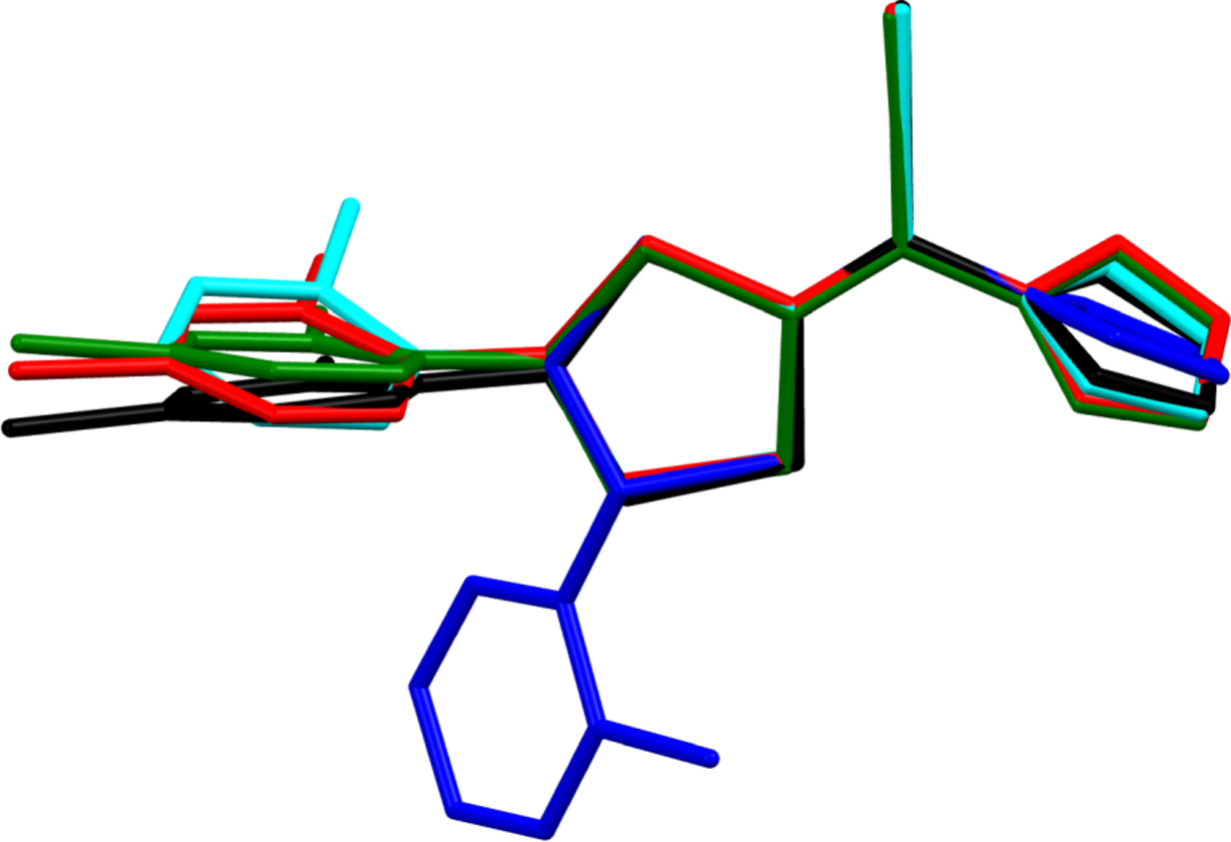
An overlay of three luliconazole mol­ecules LCZ-A (red), LCZ-B (green), LCZ-opt (black), and two related structures PESWAM (cyan) and DELYAV (blue).

The mol­ecular structure of the title compound is illustrated in Fig. 1 ▸. It crystallizes in the monoclinic crystal system, space group *P*2_1_ with one mol­ecule in the asymmetric unit. The LCZ mol­ecule has an *R* configuration at the asymmetric center of atom C8. The di­thiol­ane ring adopts an envelope conformation with the C8 flap atom having a maximum deviation of 0.287 (2) Å, and puckering parameters (Cremer & Pople, 1975 ▸) *Q* = 0.438 (2) Å and φ = 295.6 (3)°. According to the asymmetry parameters (Duax & Norton, 1975 ▸), the mirror plane passes through atom C8 and the C6—S1 bond; ΔC_s_(C8) = 10.21 (2)°. In the LCZ structure, the di­chloro­phenyl ring was found to be disordered over two orientations and the occupancies of the disordered atoms were fixed at 0.5. Fig. 2 ▸ presents an overlay of three independent luliconazole skeletons, considering separately the two disordered components A and B of LCZ, i.e. LCZ-A (red), LCZ-B (green) and the theoretically obtained optimized structure (LCZ-opt, black). The mol­ecular conformations are quite similar, differing only slightly in the orientations of the imidazole and di­chloro­phenyl rings. The dihedral angle between the disordered di­chloro­phenyl rings A and B of LCZ is 18.2 (4)°. The imidazole ring is planar (r.m.s. deviation = 0.002 Å). The mutual arrangement of the rings can be analyzed by the dihedral angles between their best planes, calculated using the least-squares method (Table 1 ▸).

## 3. Supra­molecular features

**Figure 3:**
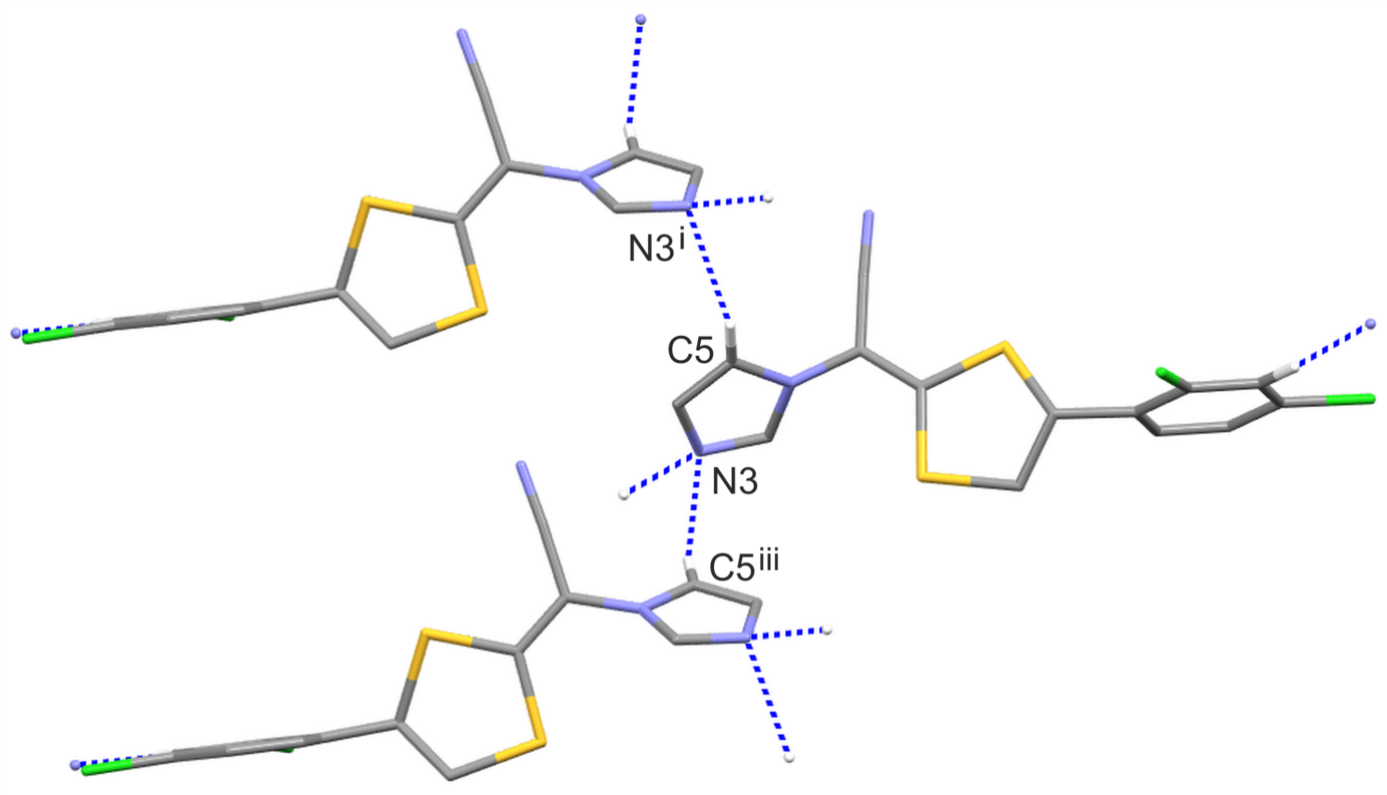
A part of the crystal structure of LCZ (only disordered component A is shown) showing the formation of the *C*(4) chain motif. Hydrogen bonds are drawn as dashed lines, and for the sake of clarity, the H atoms not involved in hydrogen bonds have been omitted. Symmetry codes: (i) −*x*, *y* + 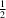, −*z*; (iii) −*x*, *y -* 1/2, −*z* + 1.

**Figure 4:**
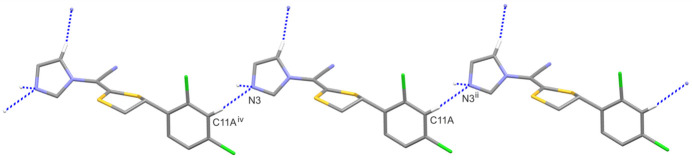
A part of the crystal structure of LCZ (only disordered component A is shown) showing the formation of the C(11) chain motif. Hydrogen bonds are drawn as dashed lines, and for the sake of clarity, the H atoms not involved in hydrogen bonds have been omitted. Symmetry codes: (ii) *x* + 1, *y*, *z* + 1; (iv) *x* − 1, *y*, *z* − 1.

**Figure 5:**
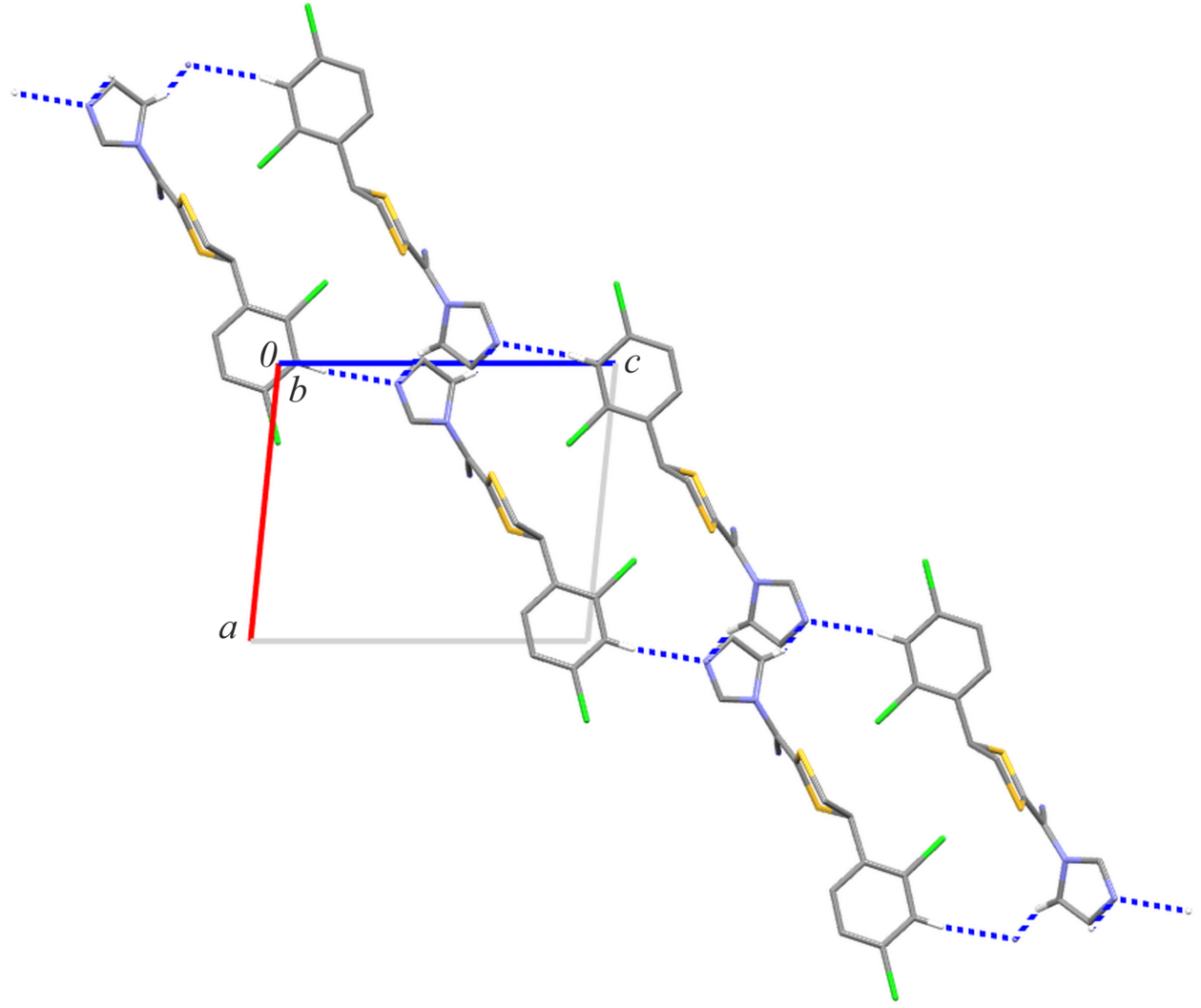
Partial crystal packing of LCZ-A showing the formation of mol­ecular layers parallel to (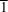
01).

In the crystal structure of luliconazole, there are no potential strong proton donors, apart from weak C—H bonds. There are two inter­molecular hydrogen bonds: C5—H5⋯N3(−*x*, *y* + 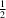, −*z* + 1) and C11*A*—H11*A*⋯N3(−*x*, *y* + 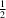, −*z* + 1); in both, the N3 atom is a hydrogen-bond acceptor. The geometric parameters of these inter­actions are presented in Table 2 ▸. Fig. 3 ▸ demonstrates that the former inter­action produces a mono-periodic chain along the *b*-axis direction, whose first-level graph-set descriptor is *C*(4) (Etter, 1990 ▸; Etter *et al.*, 1990 ▸; Bernstein *et al.*, 1995 ▸), while the latter inter­action generates a *C*(11) chain motif along the [101] direction (Fig. 4 ▸). The combination of these two chain motifs leads to the formation of di-periodic mol­ecular layers parallel to (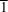
01) (Fig. 5 ▸).

## 4. Hirshfeld surface analysis

**Figure 6:**
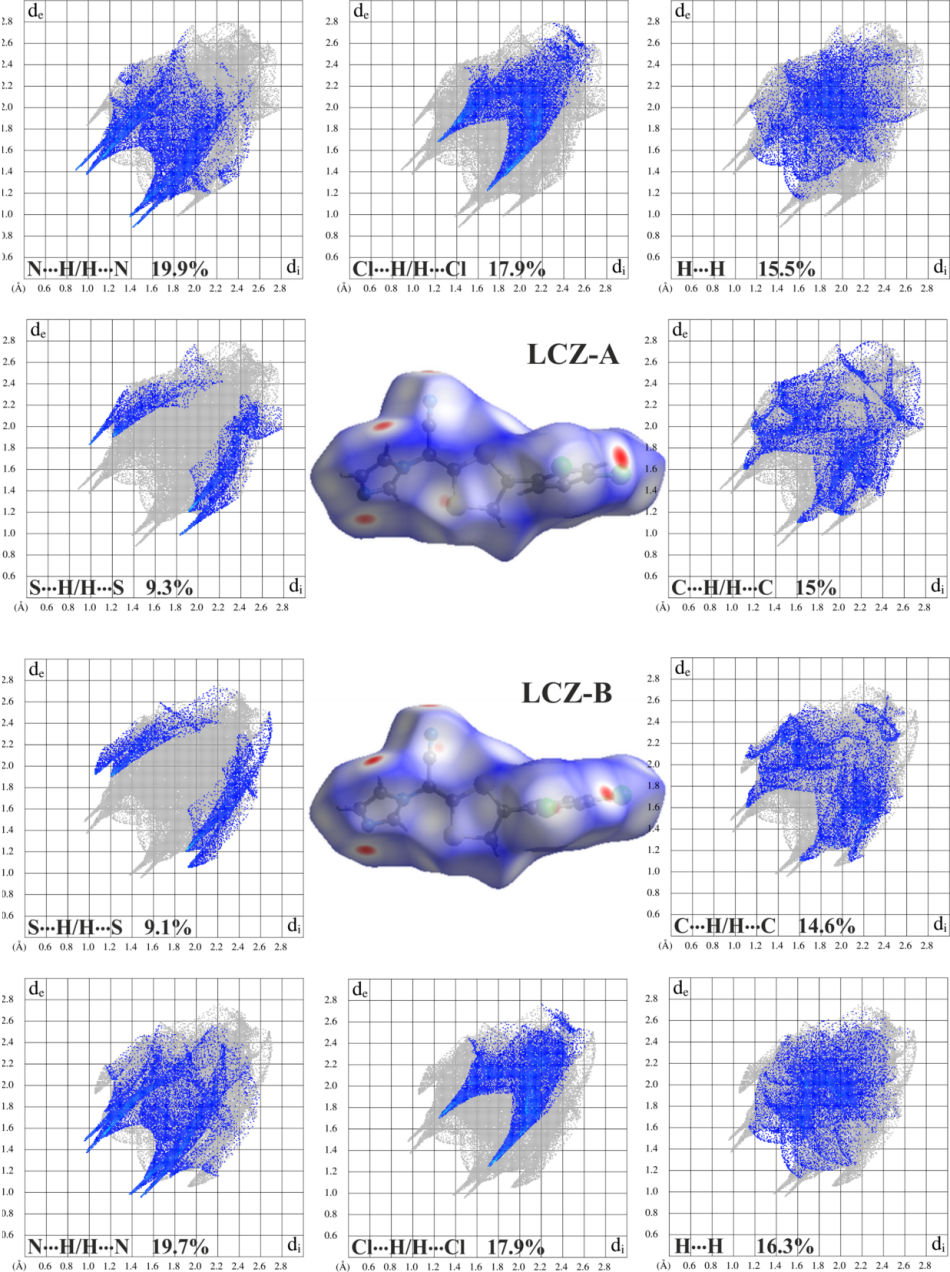
Comparison of Hirshfeld surfaces and the corresponding two-dimensional fingerprint plots of the most dominant contacts for the two disordered components of LCZ (top half for LCZ-A, bottom half for LCZ-B). The *d*_i_ and *d*_e_ values are the closest inter­nal and external distances (in Å) from given points on the Hirshfeld surface.

Hirshfeld surfaces and fingerprint plots (Spackman & McKinnon, 2002 ▸; Spackman & Jayatilaka, 2009 ▸) were generated using *CrystalExplorer* software (Spackman *et al.*, 2021 ▸). Hirshfeld surface analysis (Spackman & Jayatilaka, 2009 ▸) complements the comparison of the two disordered components of the luliconazole mol­ecule. Fig. 6 ▸ presents a comparison of the Hirshfeld surfaces of the LCZ-A and LCZ-B structures and the corresponding 2D fingerprint plots of the most dominant contacts combined with their percentage contributions to the Hirshfeld surface. Red spots on the Hirshfeld surfaces indicate atoms participating in the C—H⋯N hydrogen bonds and an N⋯S contact shorter than the sum of their van der Waals radii.

For both mol­ecules, the H⋯N/N⋯H, H⋯Cl/Cl⋯H, H⋯H and C⋯H/H⋯C contacts provide the greatest contribution (each about 15–20%) to the Hirshfeld surface. The H⋯S/S⋯H inter­actions contribute about 9%. Other contacts do not exceed 5%. Two pairs of spikes in the H⋯N/N⋯H fingerprint plots belong to the closest N⋯H contacts, while H⋯S/S⋯H inter­actions lead to characteristic sharp spikes for LCZ-A compared to the chicken-wing-like features for LCZ-B.

## 5. Database survey

A search of the Cambridge Structural Database (CSD version 5.45, June 2024, Groom *et al.*, 2016 ▸) did not reveal any structure of luliconazole. However, two isomeric structures (*E* and *Z*) were found (DELYAV; Lin *et al.*, 2006 ▸; PESWAM; Xiao *et al.*, 2006 ▸), which differ from luliconazole by the presence of a 2-chloro­phenyl ring instead of the di­chloro­phenyl ring in LCZ. Fig. 2 ▸ shows the superposition of their skeletons compared to three luliconazole mol­ecules. PESWAM (isomer *E*) is closely related in mol­ecular conformation to LCZ-A, in contrast to the structure of DELYAV, which differs from the others in the site of substitution (atom C7 *vs* C8 of the di­thiol­ane ring; isomer *Z*) and the spatial orientation of imidazole ring.

## 6. Synthesis and crystallization

The luliconazole (purity 98%) used in this study was purchased from BLD Pharmatech GmbH (Germany). A pure crystalline form of luliconazole was obtained unexpectedly from cocrystallization of the drug with pyrazinedi­carb­oxy­lic acid; all substances (0.05 mmol) were used with a fixed stoichiometric ratio of 1:1, dissolved in ethanol (3 ml EtOH) and the mixture was heated to 346 K.

## 7. Refinement

Crystal data, data collection and structure refinement details are summarized in Table 3 ▸.

During the refinement of compound LCZ, the di­chloro­phenyl ring was found to be disordered over two orientations (ring 1*A*: C9*A*, C10*A*, C11*A*, C12*A*, C13*A*, C14*A*, Cl1*A*, Cl2*A* and ring 1*B*: C9*B*, C10*B*, C11*B*, C12*B*, C13*B*, C14*B*, Cl1*B*, Cl2*B*); finally site occupancies of two components were fixed at 0.5. Two components of the disorder were modelled, using rigid planar hexa­gons for the phenyl rings. Furthermore, similarity restraints were applied to the atomic displacement parameters of all disordered atoms using SIMU and ISOR commands in *SHELXL*. The distances between atom pairs C—Cl were restrained to be equal, with an effective s.u. of 0.003 Å.

All hydrogen atoms bonded to carbon atoms were placed geometrically and refined as riding, with *U*_iso_(H) = 1.2 *U*_eq_(C) for the methyl­ene, methine and aromatic groups.

## 8. Theoretical calculations

To make a comparison between the experimental and theoretical models of luliconazole, full geometry optimization of the luliconazole mol­ecule was carried out using *GAUSSIAN16* (Frisch *et al.*, 2019 ▸) at the B3LYP/6-311++G(3df,3pd) level of theory. The input coordinates for density functional theory (DFT) calculations were generated from the experimental Cartesian coordinates of the LCZ-A structure. A stationary point of the theoretical model of luliconazole (LCZ-opt) was confirmed by the absence of imaginary frequencies. Cartesian coordinates (XYZ) for the LCZ-opt structure are given in Table S1 in the supporting information.

## Supplementary Material

Crystal structure: contains datablock(s) I, global. DOI: 10.1107/S2056989024011812/vm2310sup1.cif

Structure factors: contains datablock(s) I. DOI: 10.1107/S2056989024011812/vm2310Isup2.hkl

Cartesian coordinates (XYZ) for LCZ-opt structure. DOI: 10.1107/S2056989024011812/vm2310sup3.pdf

Supporting information file. DOI: 10.1107/S2056989024011812/vm2310Isup4.cml

CCDC reference: 2407813

Additional supporting information:  crystallographic information; 3D view; checkCIF report

## Figures and Tables

**Table 1:** Dihedral angles (°) between the best planes in LCZ-structures 1 - di­thiol­ane ring; 2 - imidazole ring; 3 - di­chloro­phenyl ring (A and B are the disordered components).

	1/2	1/3	2/3
LCZ-A	62.7 (1)	88.7 (3)	28.4 (3)
LCZ-B	62.7 (1)	74.0 (3)	46.6 (3)
LCZ-opt	81.4	75.2	36.9

**Table 2:** Hydrogen-bond geometry (Å, °)

*D*—H⋯*A*	*D*—H	H⋯*A*	*D*⋯*A*	*D*—H⋯*A*
C5—H5⋯N3^i^	0.93	2.49	3.335 (4)	151
C11*A*—H11*A*⋯N3^ii^	0.93	2.45	3.371 (6)	172
C11*B*—H11*B*⋯N3^ii^	0.93	2.58	3.440 (6)	154

Symmetry codes: (i) 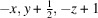; (ii) 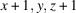.

**Table 3:** Experimental details

Crystal data	
Chemical formula	C_14_H_9_Cl_2_N_3_S_2_
*M*_r_	354.26
Crystal system, space group	Monoclinic, *P*2_1_
Temperature (K)	294
*a*, *b*, *c* (Å)	9.0136 (1), 8.1561 (1), 10.8718 (1)
β (°)	95.778 (1)
*V* (Å^3^)	795.19 (2)
*Z*	2
Radiation type	Cu *K*α
μ (mm^−1^)	6.09
Crystal size (mm)	0.20 × 0.07 × 0.03

Data collection	
Diffractometer	XtaLAB Synergy, Dualflex, HyPix
Absorption correction	Gaussian (*CrysAlis PRO*; Rigaku OD, 2023 ▸)
*T*_min_, *T*_max_	0.167, 0.787
No. of measured, independent and observed [*I* > 2σ(*I*)] reflections	14902, 3132, 3031
*R*_int_	0.026
(sin θ/λ)_max_ (Å^−1^)	0.632

Refinement	
*R*[*F*^2^ > 2σ(*F*^2^)], *wR*(*F*^2^), *S*	0.025, 0.069, 1.07
No. of reflections	3132
No. of parameters	238
No. of restraints	295
H-atom treatment	H-atom parameters constrained
Δρ_max_, Δρ_min_ (e Å^−3^)	0.15, −0.19
Absolute structure	Flack *x* determined using 1322 quotients [(*I*^+^)−(*I*^−^)]/[(*I*^+^)+(*I*^−^)] (Parsons *et al.*, 2013 ▸)
Absolute structure parameter	−0.010 (6)

Computer programs: *CrysAlis PRO* (Rigaku OD, 2023 ▸), *SHELXT* (Sheldrick, 2015*a* ▸), *SHELXL2019/2* (Sheldrick, 2015*b* ▸), *Mercury* (Macrae *et al.*, 2020 ▸), *PLATON* (Spek, 2020 ▸) and *publCIF* (Westrip, 2010 ▸).

## supplementary crystallographic information

### (E)-[(4R)-4-(2,4-Dichlorophenyl)-1,3-dithiolan-2-ylidene](1H-imidazol-1-yl)acetonitrile Crystal data

**Table:**

C_14_H_9_Cl_2_N_3_S_2_	*F*(000) = 360
*M**_r_* = 354.26	*D*_x_ = 1.480 Mg m^−^^3^
Monoclinic, *P*2_1_	Cu *K*α radiation, λ = 1.54184 Å
*a* = 9.0136 (1) Å	Cell parameters from 12732 reflections
*b* = 8.1561 (1) Å	θ = 4.1–76.6°
*c* = 10.8718 (1) Å	µ = 6.09 mm^−^^1^
β = 95.778 (1)°	*T* = 294 K
*V* = 795.19 (2) Å^3^	Prism, colourless
*Z* = 2	0.20 × 0.07 × 0.03 mm

### (E)-[(4R)-4-(2,4-Dichlorophenyl)-1,3-dithiolan-2-ylidene](1H-imidazol-1-yl)acetonitrile Data collection

**Table:**

XtaLAB Synergy, Dualflex, HyPix diffractometer	3132 independent reflections
Radiation source: micro-focus sealed X-ray tube, PhotonJet (Cu) X-ray Source	3031 reflections with *I* > 2σ(*I*)
Mirror monochromator	*R*_int_ = 0.026
Detector resolution: 10.0000 pixels mm^-1^	θ_max_ = 77.2°, θ_min_ = 4.1°
ω scans	*h* = −11→10
Absorption correction: gaussian (CrysAlisPro; Rigaku OD, 2023)	*k* = −9→10
*T*_min_ = 0.167, *T*_max_ = 0.787	*l* = −13→13
14902 measured reflections	

### (E)-[(4R)-4-(2,4-Dichlorophenyl)-1,3-dithiolan-2-ylidene](1H-imidazol-1-yl)acetonitrile Refinement

**Table:**

Refinement on *F*^2^	Hydrogen site location: inferred from neighbouring sites
Least-squares matrix: full	H-atom parameters constrained
*R*[*F*^2^ > 2σ(*F*^2^)] = 0.025	*w* = 1/[σ^2^(*F*_o_^2^) + (0.0399*P*)^2^ + 0.0887*P*] where *P* = (*F*_o_^2^ + 2*F*_c_^2^)/3
*wR*(*F*^2^) = 0.069	(Δ/σ)_max_ = 0.001
*S* = 1.07	Δρ_max_ = 0.15 e Å^−^^3^
3132 reflections	Δρ_min_ = −0.19 e Å^−^^3^
238 parameters	Absolute structure: Flack *x* determined using 1322 quotients [(*I*^+^)-(*I*^-^)]/[(*I*^+^)+(*I*^-^)] (Parsons *et al.*, 2013)
295 restraints	Absolute structure parameter: −0.010 (6)
Primary atom site location: structure-invariant direct methods	

### (E)-[(4R)-4-(2,4-Dichlorophenyl)-1,3-dithiolan-2-ylidene](1H-imidazol-1-yl)acetonitrile Special details

**Table:**

Geometry. All esds (except the esd in the dihedral angle between two l.s. planes) are estimated using the full covariance matrix. The cell esds are taken into account individually in the estimation of esds in distances, angles and torsion angles; correlations between esds in cell parameters are only used when they are defined by crystal symmetry. An approximate (isotropic) treatment of cell esds is used for estimating esds involving l.s. planes.

### (E)-[(4R)-4-(2,4-Dichlorophenyl)-1,3-dithiolan-2-ylidene](1H-imidazol-1-yl)acetonitrile Fractional atomic coordinates and isotropic or equivalent isotropic displacement parameters (Å^2^)

**Table:**

	*x*	*y*	*z*	*U*_iso_*/*U*_eq_	Occ. (<1)
Cl1A	0.7112 (8)	0.3287 (9)	1.1135 (7)	0.0901 (16)	0.5
Cl2A	1.2841 (5)	0.2715 (10)	1.0208 (5)	0.0882 (15)	0.5
Cl1B	0.7057 (9)	0.2672 (10)	1.1114 (7)	0.114 (3)	0.5
Cl2B	1.2743 (5)	0.3102 (11)	1.0307 (6)	0.105 (2)	0.5
S1	0.39907 (7)	0.21429 (7)	0.66512 (6)	0.05387 (17)	
S2	0.59952 (8)	0.49155 (9)	0.73496 (7)	0.0642 (2)	
N1	0.4012 (3)	0.8370 (3)	0.6007 (3)	0.0670 (6)	
N2	0.2121 (2)	0.4772 (3)	0.51520 (18)	0.0454 (4)	
N3	0.0737 (3)	0.3647 (3)	0.3584 (2)	0.0658 (7)	
C1	0.3416 (2)	0.5289 (3)	0.5913 (2)	0.0436 (5)	
C2	0.3726 (3)	0.6999 (3)	0.5944 (2)	0.0488 (5)	
C3	0.2085 (3)	0.3786 (3)	0.4141 (2)	0.0531 (6)	
H3	0.291839	0.327232	0.387726	0.064*	
C4	−0.0127 (3)	0.4596 (4)	0.4260 (3)	0.0724 (9)	
H4	−0.114889	0.473964	0.407721	0.087*	
C5	0.0686 (3)	0.5297 (4)	0.5224 (3)	0.0615 (7)	
H5	0.034806	0.599083	0.581389	0.074*	
C6	0.4336 (3)	0.4234 (3)	0.6568 (2)	0.0454 (5)	
C7	0.5759 (3)	0.1646 (4)	0.7514 (3)	0.0624 (7)	
H7A	0.564313	0.069235	0.802923	0.075*	
H7B	0.649020	0.138719	0.694683	0.075*	
C8	0.6294 (3)	0.3087 (4)	0.8315 (2)	0.0559 (6)	
H8	0.566602	0.317007	0.899744	0.067*	
C9A	0.7953 (5)	0.2935 (9)	0.8872 (7)	0.0553 (13)	0.5
C10A	0.8435 (7)	0.3149 (11)	1.0117 (7)	0.0556 (14)	0.5
C11A	0.9949 (8)	0.3097 (11)	1.0515 (5)	0.0628 (14)	0.5
H11A	1.027154	0.324001	1.134743	0.075*	0.5
C12A	1.0981 (5)	0.2832 (9)	0.9668 (7)	0.0683 (14)	0.5
C13A	1.0499 (6)	0.2618 (8)	0.8423 (6)	0.0619 (14)	0.5
H13A	1.118888	0.244023	0.785627	0.074*	0.5
C14A	0.8985 (7)	0.2670 (7)	0.8025 (5)	0.0579 (14)	0.5
H14A	0.866205	0.252683	0.719235	0.069*	0.5
C9B	0.7923 (5)	0.3066 (9)	0.8760 (7)	0.0528 (13)	0.5
C10B	0.8287 (7)	0.2829 (11)	1.0020 (7)	0.0566 (14)	0.5
C11B	0.9773 (8)	0.2807 (11)	1.0507 (5)	0.0646 (14)	0.5
H11B	1.001705	0.264790	1.135003	0.077*	0.5
C12B	1.0895 (5)	0.3021 (9)	0.9733 (7)	0.0632 (13)	0.5
C13B	1.0531 (6)	0.3258 (8)	0.8473 (7)	0.0648 (14)	0.5
H13B	1.128176	0.340176	0.795514	0.078*	0.5
C14B	0.9045 (7)	0.3281 (8)	0.7986 (5)	0.0602 (14)	0.5
H14B	0.880120	0.343952	0.714286	0.072*	0.5

### (E)-[(4R)-4-(2,4-Dichlorophenyl)-1,3-dithiolan-2-ylidene](1H-imidazol-1-yl)acetonitrile Atomic displacement parameters (Å^2^)

**Table:**

	*U*^11^	*U*^22^	*U*^33^	*U*^12^	*U*^13^	*U*^23^
Cl1A	0.0641 (16)	0.161 (4)	0.0466 (17)	0.009 (2)	0.0115 (11)	−0.013 (2)
Cl2A	0.0450 (13)	0.138 (4)	0.0786 (16)	0.0186 (15)	−0.0057 (10)	0.023 (2)
Cl1B	0.0780 (18)	0.213 (7)	0.051 (2)	−0.003 (3)	0.0122 (15)	0.034 (3)
Cl2B	0.0491 (16)	0.142 (4)	0.116 (3)	0.0239 (15)	−0.0284 (15)	−0.0304 (19)
S1	0.0493 (3)	0.0458 (3)	0.0639 (4)	−0.0038 (3)	−0.0072 (3)	0.0024 (3)
S2	0.0528 (3)	0.0561 (4)	0.0776 (5)	−0.0055 (3)	−0.0234 (3)	−0.0048 (3)
N1	0.0606 (14)	0.0490 (14)	0.0923 (18)	−0.0040 (11)	0.0118 (12)	−0.0051 (13)
N2	0.0347 (9)	0.0481 (11)	0.0521 (10)	−0.0016 (8)	−0.0012 (7)	−0.0022 (9)
N3	0.0610 (14)	0.0669 (16)	0.0645 (13)	−0.0120 (12)	−0.0180 (11)	0.0023 (11)
C1	0.0375 (11)	0.0453 (12)	0.0470 (11)	−0.0013 (9)	−0.0008 (9)	−0.0052 (9)
C2	0.0405 (11)	0.0481 (14)	0.0571 (13)	0.0033 (10)	0.0020 (9)	−0.0043 (11)
C3	0.0491 (13)	0.0576 (15)	0.0515 (13)	−0.0072 (11)	−0.0005 (10)	−0.0060 (11)
C4	0.0409 (13)	0.0672 (19)	0.104 (2)	−0.0011 (13)	−0.0176 (14)	0.0105 (17)
C5	0.0413 (13)	0.0551 (15)	0.087 (2)	0.0060 (11)	0.0015 (13)	−0.0022 (13)
C6	0.0436 (12)	0.0457 (12)	0.0461 (12)	−0.0020 (10)	−0.0004 (9)	−0.0065 (10)
C7	0.0556 (15)	0.0583 (17)	0.0700 (18)	0.0077 (12)	−0.0101 (13)	0.0066 (13)
C8	0.0441 (12)	0.0763 (18)	0.0460 (11)	0.0081 (13)	−0.0016 (9)	0.0004 (13)
C9A	0.047 (2)	0.077 (3)	0.042 (2)	0.006 (2)	0.002 (2)	0.003 (2)
C10A	0.050 (2)	0.076 (3)	0.039 (2)	−0.001 (2)	−0.005 (2)	−0.003 (2)
C11A	0.051 (2)	0.086 (3)	0.047 (2)	0.002 (2)	−0.013 (2)	−0.001 (2)
C12A	0.053 (2)	0.089 (3)	0.061 (2)	0.009 (2)	−0.005 (2)	0.003 (2)
C13A	0.048 (2)	0.081 (3)	0.057 (2)	0.013 (2)	0.0042 (19)	−0.002 (3)
C14A	0.050 (2)	0.078 (3)	0.045 (2)	0.008 (2)	0.0013 (18)	0.001 (2)
C9B	0.046 (2)	0.075 (3)	0.035 (2)	0.011 (2)	−0.0074 (19)	0.004 (2)
C10B	0.050 (2)	0.079 (3)	0.040 (2)	−0.001 (2)	−0.002 (2)	−0.004 (2)
C11B	0.055 (3)	0.088 (3)	0.048 (2)	0.000 (2)	−0.006 (2)	0.005 (2)
C12B	0.042 (2)	0.087 (3)	0.058 (2)	0.016 (2)	−0.006 (2)	0.001 (2)
C13B	0.049 (2)	0.085 (3)	0.060 (2)	0.010 (3)	0.005 (2)	−0.001 (3)
C14B	0.052 (2)	0.080 (3)	0.048 (2)	0.008 (3)	0.0025 (19)	0.006 (3)

### (E)-[(4R)-4-(2,4-Dichlorophenyl)-1,3-dithiolan-2-ylidene](1H-imidazol-1-yl)acetonitrile Geometric parameters (Å, º)

**Table:**

Cl1A—C10A	1.711 (3)	C7—H7B	0.9700
Cl2A—C12A	1.722 (3)	C8—C9B	1.499 (5)
Cl1B—C10B	1.710 (3)	C8—C9A	1.561 (5)
Cl2B—C12B	1.720 (3)	C8—H8	0.9800
S1—C6	1.737 (3)	C9A—C10A	1.3900
S1—C7	1.812 (3)	C9A—C14A	1.3900
S2—C6	1.736 (2)	C10A—C11A	1.3900
S2—C8	1.828 (3)	C11A—C12A	1.3900
N1—C2	1.148 (4)	C11A—H11A	0.9300
N2—C3	1.360 (3)	C12A—C13A	1.3900
N2—C5	1.371 (3)	C13A—C14A	1.3900
N2—C1	1.425 (3)	C13A—H13A	0.9300
N3—C3	1.306 (3)	C14A—H14A	0.9300
N3—C4	1.364 (4)	C9B—C10B	1.3900
C1—C6	1.348 (3)	C9B—C14B	1.3900
C1—C2	1.422 (4)	C10B—C11B	1.3900
C3—H3	0.9300	C11B—C12B	1.3900
C4—C5	1.344 (4)	C11B—H11B	0.9300
C4—H4	0.9300	C12B—C13B	1.3900
C5—H5	0.9300	C13B—C14B	1.3900
C7—C8	1.512 (4)	C13B—H13B	0.9300
C7—H7A	0.9700	C14B—H14B	0.9300
			
C6—S1—C7	95.38 (13)	C10A—C9A—C14A	120.0
C6—S2—C8	95.13 (12)	C10A—C9A—C8	124.2 (5)
C3—N2—C5	106.5 (2)	C14A—C9A—C8	115.7 (5)
C3—N2—C1	126.6 (2)	C11A—C10A—C9A	120.0
C5—N2—C1	126.6 (2)	C11A—C10A—Cl1A	121.8 (6)
C3—N3—C4	104.8 (2)	C9A—C10A—Cl1A	117.9 (5)
C6—C1—C2	120.3 (2)	C10A—C11A—C12A	120.0
C6—C1—N2	122.8 (2)	C10A—C11A—H11A	120.0
C2—C1—N2	116.8 (2)	C12A—C11A—H11A	120.0
N1—C2—C1	177.5 (3)	C13A—C12A—C11A	120.0
N3—C3—N2	111.8 (2)	C13A—C12A—Cl2A	121.5 (5)
N3—C3—H3	124.1	C11A—C12A—Cl2A	118.4 (5)
N2—C3—H3	124.1	C12A—C13A—C14A	120.0
C5—C4—N3	111.4 (2)	C12A—C13A—H13A	120.0
C5—C4—H4	124.3	C14A—C13A—H13A	120.0
N3—C4—H4	124.3	C13A—C14A—C9A	120.0
C4—C5—N2	105.4 (3)	C13A—C14A—H14A	120.0
C4—C5—H5	127.3	C9A—C14A—H14A	120.0
N2—C5—H5	127.3	C10B—C9B—C14B	120.0
C1—C6—S2	120.55 (19)	C10B—C9B—C8	116.5 (5)
C1—C6—S1	123.39 (19)	C14B—C9B—C8	123.5 (5)
S2—C6—S1	116.03 (14)	C11B—C10B—C9B	120.0
C8—C7—S1	109.7 (2)	C11B—C10B—Cl1B	113.7 (6)
C8—C7—H7A	109.7	C9B—C10B—Cl1B	126.2 (6)
S1—C7—H7A	109.7	C10B—C11B—C12B	120.0
C8—C7—H7B	109.7	C10B—C11B—H11B	120.0
S1—C7—H7B	109.7	C12B—C11B—H11B	120.0
H7A—C7—H7B	108.2	C11B—C12B—C13B	120.0
C9B—C8—C7	115.0 (4)	C11B—C12B—Cl2B	121.5 (5)
C7—C8—C9A	113.5 (3)	C13B—C12B—Cl2B	118.4 (5)
C9B—C8—S2	106.1 (3)	C14B—C13B—C12B	120.0
C7—C8—S2	106.49 (17)	C14B—C13B—H13B	120.0
C9A—C8—S2	111.6 (3)	C12B—C13B—H13B	120.0
C7—C8—H8	108.4	C13B—C14B—C9B	120.0
C9A—C8—H8	108.4	C13B—C14B—H14B	120.0
S2—C8—H8	108.4	C9B—C14B—H14B	120.0
			
C3—N2—C1—C6	−62.6 (4)	C14A—C9A—C10A—C11A	0.0
C5—N2—C1—C6	124.2 (3)	C8—C9A—C10A—C11A	−175.7 (6)
C3—N2—C1—C2	116.4 (3)	C14A—C9A—C10A—Cl1A	−174.2 (7)
C5—N2—C1—C2	−56.8 (4)	C8—C9A—C10A—Cl1A	10.0 (6)
C4—N3—C3—N2	0.6 (3)	C9A—C10A—C11A—C12A	0.0
C5—N2—C3—N3	−0.5 (3)	Cl1A—C10A—C11A—C12A	174.0 (7)
C1—N2—C3—N3	−174.8 (2)	C10A—C11A—C12A—C13A	0.0
C3—N3—C4—C5	−0.4 (4)	C10A—C11A—C12A—Cl2A	−178.1 (6)
N3—C4—C5—N2	0.1 (4)	C11A—C12A—C13A—C14A	0.0
C3—N2—C5—C4	0.2 (3)	Cl2A—C12A—C13A—C14A	178.0 (6)
C1—N2—C5—C4	174.5 (3)	C12A—C13A—C14A—C9A	0.0
C2—C1—C6—S2	−5.7 (3)	C10A—C9A—C14A—C13A	0.0
N2—C1—C6—S2	173.31 (18)	C8—C9A—C14A—C13A	176.1 (6)
C2—C1—C6—S1	176.36 (19)	C7—C8—C9B—C10B	−111.8 (4)
N2—C1—C6—S1	−4.7 (3)	S2—C8—C9B—C10B	130.8 (3)
C8—S2—C6—C1	164.4 (2)	C7—C8—C9B—C14B	68.7 (6)
C8—S2—C6—S1	−17.54 (17)	S2—C8—C9B—C14B	−48.7 (5)
C7—S1—C6—C1	174.3 (2)	C14B—C9B—C10B—C11B	0.0
C7—S1—C6—S2	−3.76 (18)	C8—C9B—C10B—C11B	−179.5 (6)
C6—S1—C7—C8	29.9 (2)	C14B—C9B—C10B—Cl1B	175.7 (7)
S1—C7—C8—C9B	−161.5 (4)	C8—C9B—C10B—Cl1B	−3.8 (7)
S1—C7—C8—C9A	−167.4 (4)	C9B—C10B—C11B—C12B	0.0
S1—C7—C8—S2	−44.3 (2)	Cl1B—C10B—C11B—C12B	−176.2 (7)
C6—S2—C8—C9B	159.7 (3)	C10B—C11B—C12B—C13B	0.0
C6—S2—C8—C7	36.8 (2)	C10B—C11B—C12B—Cl2B	176.8 (7)
C6—S2—C8—C9A	161.1 (3)	C11B—C12B—C13B—C14B	0.0
C7—C8—C9A—C10A	−129.6 (4)	Cl2B—C12B—C13B—C14B	−176.9 (6)
S2—C8—C9A—C10A	110.0 (4)	C12B—C13B—C14B—C9B	0.0
C7—C8—C9A—C14A	54.5 (5)	C10B—C9B—C14B—C13B	0.0
S2—C8—C9A—C14A	−65.9 (5)	C8—C9B—C14B—C13B	179.5 (6)

### (E)-[(4R)-4-(2,4-Dichlorophenyl)-1,3-dithiolan-2-ylidene](1H-imidazol-1-yl)acetonitrile Hydrogen-bond geometry (Å, º)

**Table:**

*D*—H···*A*	*D*—H	H···*A*	*D*···*A*	*D*—H···*A*
C5—H5···N3^i^	0.93	2.49	3.335 (4)	151
C11*A*—H11*A*···N3^ii^	0.93	2.45	3.371 (6)	172
C11*B*—H11*B*···N3^ii^	0.93	2.58	3.440 (6)	154

Symmetry codes: (i) −*x*, *y*+1/2, −*z*+1; (ii) *x*+1, *y*, *z*+1.
