# Supplementary material for: Crystal structure of luliconazole
Source: Acta Crystallogr E Crystallogr Commun. 2025 Jan 1;81(Pt 1):24–8. doi: 10.1107/S2056989024011812 (PMC11701779; doi:10.1107/S2056989024011812)
Supplement: Supplementary file 3 [file e-81-00024-sup3.pdf]

Supporting information

Table S1. Final Cartesian coordinates (X, Y, Z in Å) for the gas-phase structure of LCZ-opt optimized at the B3LYP/6-311++G(3df,3pd) level of theory.

Atom	X	Y	Z
Cl	-2.790654	2.385390	1.332457
Cl	-6.423827	-1.076077	-0.567068
S	1.800501	1.129939	-1.008332
S	0.411040	-0.863954	0.692393
N	3.324574	-2.540733	2.301134
N	4.343589	-0.199299	-0.042846
N	6.044500	0.033043	-1.448089
C	3.132969	-0.692680	0.518811
C	3.236476	-1.712500	1.501374
C	4.910355	-0.575786	-1.236274
H	4.434164	-1.291167	-1.886241
C	6.246789	0.837050	-0.349087
H	7.120973	1.459200	-0.260536
C	5.215906	0.709129	0.534156
H	5.012861	1.155323	1.490836
C	1.928653	-0.207523	0.119622
C	-0.000975	0.992689	-1.240022
H	-0.368303	1.978791	-1.522690
H	-0.210286	0.291718	-2.046026
C	-0.628611	0.534092	0.075473
H	-0.538284	1.329598	0.808988
C	-2.076082	0.126817	-0.056715
C	-3.114952	0.903302	0.466701
C	-4.448229	0.541835	0.314661
H	-5.228644	1.160011	0.730766
C	-4.758185	-0.618327	-0.376555
C	-3.756295	-1.415327	-0.914591
H	-4.006357	-2.319963	-1.448519
C	-2.435830	-1.033635	-0.747810
H	-1.655163	-1.663466	-1.153028
